# Small and inconsistent effects of whole body vibration on athletic performance: a systematic review and meta-analysis

**DOI:** 10.1007/s00421-015-3194-9

**Published:** 2015-06-03

**Authors:** Tibor Hortobágyi, Melanie Lesinski, Miguel Fernandez-del-Olmo, Urs Granacher

**Affiliations:** Center for Human, Movement Sciences, University of Groningen, University Medical Center Groningen, A. Deusinglaan 1, 9700 AD Groningen, The Netherlands; Faculty of Health and Life Sciences, Northumbria University, Newcastle upon Tyne, UK; Division of Training and Movement Sciences, University of Potsdam, Potsdam, Germany; Learning and Human Movement Control Group, University of A Coruña, A Coruña, Spain

**Keywords:** Exercise, Muscle, Force, Power, Skill, Reflex, Endocrine, Metabolism

## Abstract

**Purpose:**

We quantified the acute and chronic effects of whole body vibration on athletic performance or its proxy measures in competitive and/or elite athletes.

**Methods:**

Systematic literature review and meta-analysis.

**Results:**

Whole body vibration combined with exercise had an overall 0.3 % acute effect on maximal voluntary leg force (−6.4 %, effect size = −0.43, 1 study), leg power (4.7 %, weighted mean effect size = 0.30, 6 studies), flexibility (4.6 %, effect size = −0.12 to 0.22, 2 studies), and athletic performance (−1.9 %, weighted mean effect size = 0.26, 6 studies) in 191 (103 male, 88 female) athletes representing eight sports (overall effect size = 0.28). Whole body vibration combined with exercise had an overall 10.2 % chronic effect on maximal voluntary leg force (14.6 %, weighted mean effect size = 0.44, 5 studies), leg power (10.7 %, weighted mean effect size = 0.42, 9 studies), flexibility (16.5 %, effect size = 0.57 to 0.61, 2 studies), and athletic performance (−1.2 %, weighted mean effect size = 0.45, 5 studies) in 437 (169 male, 268 female) athletes (overall effect size = 0.44).

**Conclusions:**

Whole body vibration has small and inconsistent acute and chronic effects on athletic performance in competitive and/or elite athletes. These findings lead to the hypothesis that neuromuscular adaptive processes following whole body vibration are not specific enough to enhance athletic performance. Thus, other types of exercise programs (e.g., resistance training) are recommended if the goal is to improve athletic performance.

## Introduction

Human responses to mechanical vibrations have been widely studied since the middle of the nineteenth century (Taylor 1880). The initial studies in the fields of occupational therapy and work physiology considered mechanical vibration as an environmental stressor: many blue-collar workers exposed to chronic mechanical vibration reported vertigo, motion sickness, and low back pain (Helmkamp et al. 1984). Concurrently and somewhat paradoxically, sport scientists as early as 1978 also started to explore the application of mechanical vibration as a potential stimulus for increasing muscle function and athletic performance (Bosco et al. 1998, 1999b; Issurin et al. 1994; Mester et al. 1999; Nazarov and Spivak 1985; Zagorskaia 1978). The reader is referred to a previous review that provides a comprehensive description of whole body vibration currently used by athletes, including device types, physics principles, frequency, amplitude, acceleration, muscle and tendon mechanics, and neuronal and physiological responses (Rittweger 2010).

The results of these early studies were inconsistent. For example, there were reports of up to 50 % increases in the one-repetition-maximum (1RM) muscle strength measured during seated bench pull after 3 weeks of strength training with superimposed vibration in male physical education students age 19–25 (Issurin et al. 1994). In contrast, there were no improvements in isometric and dynamic knee extensor and flexor strength following 5 weeks of whole body vibration that was conducted prior to the conventional training program in sprint-trained athletes’ age 17–30 (Delecluse et al. 2005). A systematic review also reported that four of five studies included in the analysis revealed no differences in strength improvements produced by exercise training with and without whole body vibration (Nordlund and Thorstensson 2007). Notwithstanding the availability of such and other relevant information (Cardinale and Erskine 2008; Cardinale and Wakeling 2005; Cormie et al. 2006; Mansfield 2005), the number of studies exploring the effects of whole body vibration on the ability to generate maximal voluntary force and power has grown exponentially over the past decade in the lay and scientific literature.

At the highest level of athletic performance, it is difficult to provide an extra training stimulus that has a singular or an additive effect to the currently used training volume and intensity. Elite athletes carefully calibrate and plan the nature of the physical conditioning training stimulus, which nears or is already at a maximal level in the form of strength or power training. Therefore, a particularly acute issue is whether whole body vibration, as an adjuvant training modality, could improve competitive athletes’ neuromuscular function. Despite the immense popularity of the topic reflected by the dozens of whole body vibration studies published annually, the question whether or not whole body vibration could increase athletic performance was last reviewed nearly a decade ago (Cardinale and Erskine 2008; Issurin 2005). A systematic re-analysis of the literature appears timely because these and other previous reviews included subjects with different levels of physical fitness, from sedentary old adults to young ballerinas (Manimmanakorn et al. 2014).

Previous analytical reviews identified several potential mechanisms of how whole body vibration may exert its beneficial effects on neuromuscular function in general and on athletic performance in particular (Nordlund and Thorstensson 2007; Rittweger 2010). These mechanisms included an acute force enhancement through the vibration-induced rapid stretch-shortening cycle, facilitation of muscle function through the tonic vibration reflex, an enhancement of muscle energy metabolism through vibration-generated muscle contraction, increases in muscle perfusion rates, rise in muscle temperature, and favorable changes in growth hormone, testosterone, and catecholamine levels (Nordlund and Thorstensson 2007; Rittweger 2010). However, a recent scoping review that qualitatively analyzed these and other effects specifically on athletic performance arrived at a conclusion that it is unlikely that any of these mechanisms would be robust enough to add to the omnibus effects provided by the primary training stimulus (Hortobágyi et al. 2014). This scoping review served as a qualitative basis for the present quantitative analyses.

The purpose of the present review and meta-analysis was to statistically quantify any potential systematic effects that whole body vibration may have on athletic performance and on some of its proxy measures in competitive and/or elite athletes. Based on preliminary analyses (Hortobágyi et al. 2014; Nordlund and Thorstensson 2007), we expected that when combined with the main exercise stimulus, whole body vibration would provide little or no systematic extra effects on athletic performance. We based this hypothesis on the observations that: (1) most studies administered the whole body vibration stimulus off-season and assumed that the extra effects would transfer to the competitive phase when the quantity and quality of the training stimulus is different; (2) the whole body vibration stimulus has low or no specificity to the structure of the motor skills involved in a given sport, questioning the ecological validity of the results, and (3) the magnitude of stimulus provided by whole body vibration in relation to the magnitude of stimulus generated by physical conditioning and skill training is so small that it would have minimal or no performance-enhancing effects.

## Methods

### Literature search

We performed a computerized systematic literature search in PubMed, Web of Science, and SportDiscus from January 1984 up to March 2015. We used the following Boolean Search Strategy in PubMed—((Vibration*[MeSH Terms] OR Vibration*[All Fields]) AND (athlete*[MeSH Terms] OR athlete*[All Fields])) with the following filters activated: Text availability: full text; Publication dates: 1 January 1984 to 31 March 2015; Species: humans, Ages: adolescent: 13–18 years, adult: 19–44 years; Languages: English. The syntax in the Web of Science and SportDiscus was as follows: (Vibration*) AND (athlete* OR elite) NOT (old* OR patient* OR elder* OR aged). Filters were additionally activated to focus the search on articles published in academic journals in English. We scanned the reference lists of each included article and also the references of relevant reviews to identify additional suitable studies for inclusion in the database.

### Selection criteria

We included only studies that examined elite and/or competitive athletes, i.e., experts in their discipline, who participate in high performance exercise training and have already achieved national and/or international level in their discipline (Lesinski et al. 2013, 2014). We excluded studies that used ‘recreational athletes’, ‘physical education students’, ‘undergraduate dance majors’, ‘sports club’, ‘amateur’, ‘non-competitive’ athletes, college level athletes in the USA lower than Division I, and ‘athletes’ without specifying the level of standard. We refer to a few of these excluded studies without aiming for a comprehensive list (Bertuzzi et al. 2013; Cochrane and Booker 2014; Fachina et al. 2013; Jones et al. 2011; Reyes et al. 2011; Ronnestad and Ellefsen 2011; Viru et al. 2007; Wyon et al. 2010). We excluded studies that did examine ‘competitive’ and/or ‘elite athletes’ but did not use a comparison control group or a control condition (Bosco et al. 1999b; Bunker et al. 2011; Roberts et al. 2009; Ronnestad et al. 2012, 2013), had no baseline data (Crow et al. 2012; Dabbs et al. 2010) or it was not possible to extract the means and standard deviations (Crow et al. 2012; Jones 2014; Mester et al. 2006), the athletes were outside the age range of 13–44 years (Kinser et al. 2008; Mahieu et al. 2006) to represent elite performance levels or measured features of athletic performance (i.e., gait kinematics) but not performance itself (Padulo et al. 2014). We also excluded studies that applied vibration focally directly to the muscle belly or tendon, through probes, hand-held devices, or cables (Bosco et al. 1999a; Cochrane and Hawke 2007; Issurin and Tenenbaum 1999) and used 50 Hz or higher frequencies (Bunker et al. 2011). In summary, we examined the acute and chronic effects of whole body vibration on (a) maximal voluntary force and power as proxies for athletic performance; (b) flexibility and joint range of motion; and (c) on athletic performance (e.g., basketball, sprinting, field hockey, netball, etc.). Based on inclusion and exclusion criteria, two independent reviewers (ML, UG) screened potentially relevant papers based on title and abstract and then the full text of candidate articles was reviewed for eligibility.

### Coding of studies

Each study was coded for the following variables: age, sex, number, and competition level of athletes, and sport they pursued. We coded whole body vibration for type, frequency, amplitude, acceleration, and duration of exposure time and in the chronic studies, also for training period and training frequency. We extracted the main features in which whole body vibration was applied in its acute form and characterized essential parameters of chronic interventions. We paid special attention to determine the nature of the control conditions and control intervention, i.e., whether or not it was active or passive. For example, standing on the whole body vibration platform in a semi-squat akimbo position with and without (i.e., “sham condition”) the vibration turned on would represent a comparison between whole body vibration and a passive control condition. In contrast, performing squats with 100 kg mass with and without the vibration turned on would represent a comparison between whole body vibration and an active control. For a given study, there were often multiple outcome measures, qualifying the study for inclusion in more than one of the four performance categories (i.e., MVC, power, flexibility, athletic performance). We included one outcome measure from a given study within one performance category. Although several authors were helpful and responded to our request for the data, we included one study in which we estimated the pre- and post-testing means and standard deviations from the published figures (Lovell et al. 2013).

### Assessment of methodological quality and statistical analyses

We used the Physiotherapy Evidence Database (PEDro) scale to quantify the quality of the eligible studies. The PEDro Scale rates internal study validity and the presence of statistically replicable information on a scale from 0 to 10 with 6 representing a cutoff score for high-quality studies (Maher et al. 2003).

To determine the effectiveness of whole body vibration on an outcome measure, we computed between-subject effect size as follows: effect size = ± ([mean post-value intervention group − mean post-value control group]/pooled variance). Using Review Manager version 5.3.4, we adjusted the effect size for sample size. In addition, weighting of the studies was applied in Review Manager version 5.3.4 according to the magnitude of the respective standard error. Because several studies did not use a control group but instead used a control (no treatment) vs. experimental condition comparison in the same subjects, we used a random effect meta-analysis model in Review Manager to compute weighted mean effect sizes (Higgins et al. 2003). Depending on a given outcome measure (i.e., sprint time vs. sprint speed), effect size can be negative or positive. For the sake of consistency and to improve readability, we report positive changes in outcomes and superiority of whole body vibration compared with control. The calculation of effect size makes it possible to conduct a systematic and quantitative evaluation whether or not acute and chronic whole body vibration vs. control interventions affects MVC force, power, and athletic performance and if so, whether these differences are also of practical importance. Effect size values of 0.00 ≤ 0.49 indicate small, 0.50 ≤ 0.79 indicate medium, and ≥0.80 indicate large practical effects (Cohen 1988).

## Results

### Study characteristics

**Fig. 1:**
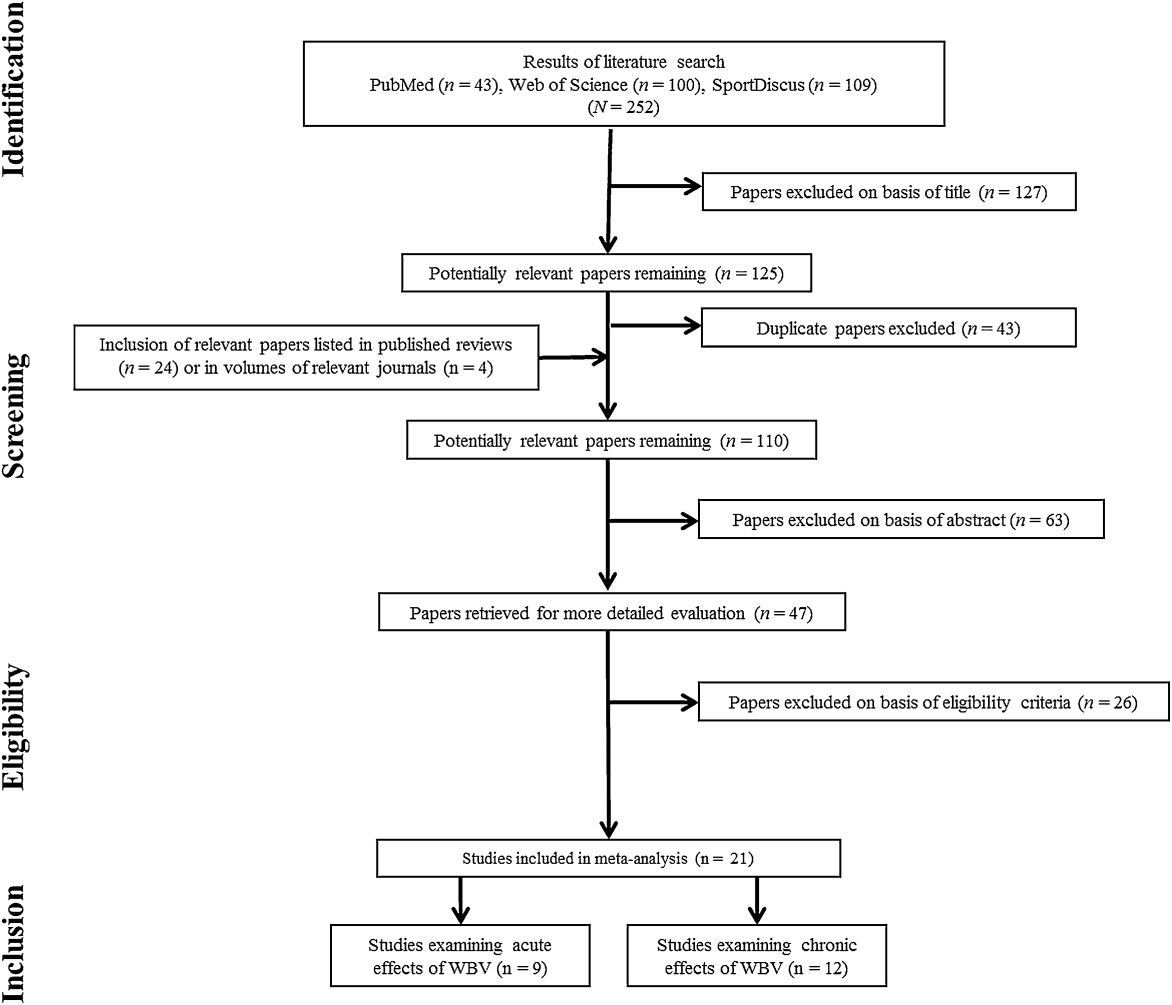
Flowchart illustrating the different phases of the search and study selection

Figure 1 shows the study selection flow chart. The search identified a total of 21 eligible studies from an original search yield of 252 studies. Our original intention was to determine the acute and chronic effects of whole body vibration on four outcome measures: maximal voluntary force, power, flexibility, and athletic performance. However, the search identified only one study that examined the acute effects of whole body vibration on maximal voluntary force (Lovell et al. 2013) and two studies that examined, respectively, the acute (Cochrane and Stannard 2005; Despina et al. 2014) and chronic (Fagnani et al. 2006; Marshall and Wyon 2012) effects of whole body vibration on flexibility. Because pooling effect size in such low number of studies has the potential for a biased conclusion, we did not pool effect sizes for these studies in these outcome categories (i.e., maximal voluntary force and flexibility) but these studies were still included in the forest plots in other outcome categories (power, athletic performance).

Tables 1 and 2 show the characteristics of the 21 studies included in the analyses. There were 373 (173 male, 200 female) athletes’ age 22.1 years (±SD 2.6, range 17.0–27.0), representing 16 sports. Of the 21 studies, nine examined the acute effects of whole body vibration on selected outcome measures in 125 (82 male, 43 female) athletes, representing eight unique sports and 12 studies examined the chronic effects of whole body vibration on selected outcomes in 248 (91 male, 157 female) athletes, representing eight unique sports. We retained the authors’ original description concerning the athletes’ level of competition. Acute and chronic studies used, respectively, similar whole body vibration frequency (33.6 vs. 32.5 Hz, *p* = 0.659), amplitude (3.2 vs. 3.8 mm, *p* = 0.201), and acceleration (126 vs. 181 m/s^2^, *p* = 0.132), resulting in an overall frequency of 33.0 (±5.2, range 25–45) Hz, amplitude of 3.8 (±1.8, range 0.8–8.0) mm, and acceleration of 157 (±82, range 52–386) m/s^2^ in the 21 studies. In the acute studies (Table 1), the average duration of exposure to vibration was 145.0 s (±112.9, range 20–300). In the chronic studies (Table 2), the average duration of exposure to vibration was 107.7 min (±52.7, range 30–189). Table 3 shows the quality of the acute and chronic studies. The mean values of 5.7 (acute) and 4.5 (chronic) PEDro scores were below the recommended threshold of 6.0 (Maher et al. 2003).

**Table 1:** Characteristics of studies that examined the acute effects of whole body vibration on selected performance outcomes in competitive/elite athletes

References	Age, years	Sport	Level	*n*	*n*, M	*n*, F	WBV type	*f*, Hz	*A*, mm	*a*, m/s^2^	*t*, s	Parameter, measure	Intervention
Bullock et al. (2008)	25 ± 5	Skeleton	International	7	1	6	Nemes, vertical sinusoid	30	4.0 pp	142	180	CMJ [cm]; 30 m split sprint time [s]	EG: WBV + squat; CG: passive control (unloaded half squat)
Cochrane and Stannard (2005)	22 ± 6	Field hockey	Elite	18	0	18	Galileo, vertical side-alternating	26	6.0 pp	160	300	CMJ [cm]; sit and reach [cm]	EG: WBV; CGI: active control (control cycling); CGII: passive control
Cochrane (2013)	20 ± 1	Netball	Premier	8	0	8	Galileo, vertical side-alternating	26	6.0 pp	160	300	5 m sprint time [s]; reactive agility sprint [s]	EG: WBV + half squats no extra exercise; CG: passive control (half squats)
Despina et al. (2014)	18 ± 1	Rhytmic gymn	Olympic	11	0	11	Galileo, vertical side-alternating	30	2.0 nr	71	75	CMJ [cm]; weight shift [%]; balance at 1 and 15 min. post [%]	EG: WBV + squats; CG: active control (squats)
Guggenheimer et al. (2009)	EG + CGI: 21 ± 3	Track and field	Competitive college	23	23	0	Pneu-Vibe Pro	30	1.5 nr	53	20	40 m sprint [s]	EG: WBV + high-knee running; CGI: active control (high-knee running); CGII: active control (power clean)
	CGII: 20 ± 1												
Lovell et al. (2013)	20 ± 1	Soccer	Semi-pro	10	10	0	PowerPlate, vertical sinusoid	40	0.83 pp	52	180	CMJ [cm]; 10 m sprint [s]; concentric MVC [Nm]; eccentric MVC [Nm]	EG: WBV; CGI: active control (control agility); CGII: passive control
Naclerio et al. (2014)	20 ± 1	Football, baseball	College	15	15	0	PowerPlate, vertical sinusoid	40	1.96 pp	126	nr	CMJ [W]; drop jump [W]	EG: WBV + 80 % 1RM squat; CGI: active control (80 % 1RM squat); CGII: passive control
Padulo et al. (2014)	17 ± 0.5	Soccer	National	17	17	0	PowerPlate, vertical sinusoid	45	2.2 pp	176	75	40 m shuttle run [s]	EG: WBV + 6 × 40 m shuttle runs; CGI: active control (run 1); CGII: active control (run 3)
Rhea and Kenn (2009)	23 ± 4	Track and field	College	16	16	0	iTonic platform	35	4.0 nr	193	30	75 % RM squat	EG: WBV + squats; CG: passive control

*A* amplitude, *a* acceleration, *CG* control group I or II, *CMJ* countermovement jump, *DJ* drop jump, *Ecc* eccentric contraction, *EG* experimental group, *F* female, *f* frequency, *Iso* isometric contraction, *M* male, *MVC* maximal voluntary contraction, *n* number of subjects, *nr* not reported, *pp* peak to peak amplitude, *RM* repetition maximum, *ROM* range of motion, *ST* *training* strength training, *t* total whole body vibration exposure time, *WBV* whole body vibration

**Table 2:** Characteristics of studies that examined the chronic effects of whole body vibration on selected performance outcomes in competitive/elite athletes

References	Age, years	Sport	Level	*n*	*n*, M	*n*, F	WBV type	*f*, Hz	*A*, mm	*a*, m/s^2^	*t*, min	Intervention duration, frequency	Intervention	Parameter, measure	Intervention
Annino et al. (2007)	21 ± 2	Dance	National	22	0	22	Nemes, vertical sinusoid	30	5.0 nr	177	80	8 weeks; 3/week	EG: WBV + unloaded half squat; CG: passive control (unloaded half squats)	CMJ [cm]; leg press at 100 kg [W]	EG: 8 weeks WBV + unloaded half squat; CG: passive control (8 weeks unloaded half squats)
Cheng et al. (2012)	20 ± 2	Mix (volleyball, tennis, taekwondo, track)	Competitive college	24	24	0	Body Green Platform, vertical	30	1.5 nr	53	180	8 weeks; 3/week	EG: WBV + half squats no extra exercise; CG: passive control (half squats no extra exercise)	Running economy [m/ml/kg]; Maximal rate of tension development [N/ms]	EG: 8 weeks WBV + half squats no extra exercise; CG: passive control (8 weeks half squats no extra exercise)
Colson et al. (2010)	EG: 20 ± 2	Basketball	Competitive regional	18	13	5	Silverplate, vertical	40	4.0 pp	252	120	4 weeks; 3/week	EG: WBV + unloaded squat; CG: passive control	CMJ [cm]; 30 s jump [W/kg]; Isometric MVC [N]	EG: 4 weeks WBV + unloaded squat; CG: passive control
	CG: 19 ± 1														
Delecluse et al. (2005)	EG-M: 22 ± 3	Sprinting	Elite	20	13	7	PowerPlate, vertical sinusoid	35	2.0 nr	97	189	5 weeks; 3/week	EG: WBV + sprint; CG: active control (sprint)	30 m sprint start time [ms]; 30 m sprint start velocity [m/s]	EG: 5 weeks WBV + sprint; CG: active control (5 weeks sprint)
	EG-F: 20 ± 2														
	CG-M: 21 ± 4														
	CG-F: 23 ± 6														
Fagnani et al. (2006)	24 ± 2	Mix (volleyball, track, basketball, gymn)	Elite	24	0	24	Nemes, vertical sinusoid	25	4.0 nr	99	82.5	8 weeks; 3/week	EG: WBV + practice own discipline; CG: active control (practice own discipline)	3RM leg press [kg]; CMJ [cm]; sit and reach [cm]	EG: 8 weeks WBV + practice own discipline; CG: active control (8 weeks practice own discipline)
Fernandez-Rio et al. (2010)	17 ± 5	Basketabll	Professional and junior	31	0	31	PowerPlate, vertical sinusoid	35	4.0 nr	193	69	14 weeks; 2/week	EG: WBV + unloaded squat, toe standing; CG: passive control (unloaded squats, toe standing)	CMJ [ms]; squat power [W]	EG: 14 weeks WBV + unloaded squat, toe standing; CG: passive control (unloaded squats, toe standing)
Fernandez-Rio et al. (2012)	EG: 24 ± 3	Basketball	Inter/national	10	0	10	PowerPlate, vertical sinusoid	35	4.0 nr	193	108-162	12 weeks; 3/week	EG: WBV + unloaded squats, toe standing; CG: passive control (unloaded squats, toe standing)	CMJ [ms]; 15 s jump [W/kg]	EG: 12 weeks WBV + unloaded squats, toe standing; CG: passive control (unloaded squats, toe standing)
	CG: 23 ± 4														
Fort et al. (2012)	16 ± 1	Basketball	Competitive	23	0	23	Nemes, vertical sinusoid	30	4.0 nr	142	nr	15 weeks; nr	EG: WBV + squat, step, jump program; CG: active control (squat, step, jump program)	CMJ [cm]; right leg standing balance [s]; left leg standing balance [s]	EG: 15 weeks WBV + squat, step, jump program; CG: active control (15 weeks squat, step, jump program)
Marshall and Wyon (2012)	EG: 22 ± 1	Dance	College	17	0	17	Unknown	35	8.0 pp	386	84	4 weeks; 2/week	EG: WBV + 9 dance exercises; CG: active control (9 dance exercises)	CMJ [cm]; right hip ROM [°]	EG: 4 weeks WBV + 9 dance exercises; CG: active control (4 weeks 9 dance exercises)
	CG: 25 ± 6														
Preatoni et al. (2012)	EGI: 26 ± 5	Mix (soccer, softball)	National	18	0	18	Nemes, vertical sinusoid	35	6.0 pp	290	nr	8 weeks; 2/week	EGI: WBV + ST training; EGII: WBV; CG: active control (ST training)	Isometric MVC [N]; 15 s jump [W/kg]	EGI: 8 weeks WBV + strength training; EGII: 8 weeks WBV; CG: active control (8 weeks strength training)
	EGII: 24 ± 5														
	CG: 22 ± 2														
Suarez-Arrones et al. (2014)	27 ± 2	Rugby	Semi-pro	20	20	0	Custom design, Vfsport, vertical	30	4.0 pp	142	nr	6 weeks; 1/week	EG: WBV + shuttle run + ST training; CG: active control (shuttle run training)	Loaded explosive squats [W]; 3 × 20 m repeated sprint [s]	EG: 6 weeks WBV + shuttle run + strength training; CG: active control (6 weeks shuttle run training)
Wang et al. (2014)	21 ± 2	Sprinting	National	21	21	0	Custom design, Magtonic, vertical	30	4.0 pp	142	30	4 weeks; 3/week	EGI: WBV + 75 % MVC ST; EGII: WBV; CG: active control (75 % MVC ST)	30 m speed [m/s]; Isometric MVC [Nm/kg]; Eccentric MVC [Nm/kg]	EGI: 4 weeks, WBV + 75 % MVC strength training; EGII: 4 weeks, WBV; CG: active control (4 weeks, 75 % MVC strength training)

*A* amplitude, *a* acceleration, *CG* control group I or II, *CMJ* countermovement jump, *DJ* drop jump, *Ecc* eccentric contraction, *EG* experimental group, *F* female, *f* frequency, *Iso* isometric contraction, *M* male, *MVC* maximal voluntary contraction, *n* number of subjects, *nr* not reported, *pp* peak to peak amplitude, *RM* repetition maximum, *ROM* range of motion, *ST training* strength training, *t* total whole body vibration exposure time, *WBV* whole body vibration

**Table 3:** Physiotherapy evidence database (PEDro) scores of the reviewed acute (top panel) and chronic whole body vibration studies (lower panel)

Acute studies	Eligibility criteria specified	Random allocation	Allocation concealed	Groups similar at baseline	Blinding of subjects	Blinding of therapists	Blinding of assessors	Dropout <15 %	Intention-to-treat method	Statistical b/w-group comparison	Point measures, measures of variability	PEDro score
Bullock et al. (2008)	−	+	−	−	−	−	−	+	+	+	+	5
Cochrane and Stannard (2005)	−	+	−	+	−	−	−	+	+	+	+	6
Cochrane (2013)	+	+	−	−	−	−	−	+	+	+	+	5
Despina et al. (2014)	+	+	−	−	−	−	−	+	+	+	+	6
Guggenheimer et al. (2009)	+	+	+	+	−	−	−	+	+	+	+	7
Lovell et al. (2013)	+	+	−	+	−	−	−	+	+	+	+	6
Naclerio et al. (2014)	+	+	−	−	−	−	−	+	+	+	+	5
Padulo et al. (2014)	+	−	−	+	−	−	−	+	+	+	+	5
Rhea and Kenn (2009)	+	+	−	+	−	−	−	+	+	+	+	6
											Mean	5.7

Chronic studies	Eligibility criteria specified	Random allocation	Allocation concealed	Groups similar at baseline	Blinding of subjects	Blinding of therapists	Blinding of assessors	Dropout <15 %	Intention-to-treat method	Statistical b/w-group comparison	Point measures, measures of variability	PEDro score
Annino et al. (2007)	+	+	−	+	−	−	−	−	−	+	+	4
Cheng et al. (2012)	−	+	−	−	−	−	−	+	+	+	+	5
Colson et al. (2010)	+	+	−	+	−	−	−	+	+	+	+	6
Delecluse et al. (2005)	+	+	−	+	−	−	−	−	−	+	+	4
Fagnani et al. (2006)	+	+	−	+	−	−	−	+	+	+	+	6
Fernandez-Rio et al. (2010)	−	+	−	−	−	−	−	+	−	+	+	4
Fernandez-Rio et al. (2012)	−	−	−	+	−	−	−	−	−	+	+	3
Fort et al. (2012)	+	+	−	+	−	−	−	+	−	+	+	5
Marshall and Wyon (2012)	+	+	−	−	−	−	−	−	−	+	+	3
Preatoni et al. (2012)	+	+	−	+	−	−	−	+	−	+	+	5
Suarez-Arrones et al. (2014)	+	−	−	+	−	−	−	−	−	+	+	3
Wang et al. (2014)	+	+	−	+	−	−	−	+	+	+	+	6
											Mean	4.5

### Effects of whole body vibration on maximal voluntary force in athletes

#### Acute effects

The search identified one study that examined the acute effects of whole body vibration on maximal voluntary force (Lovell et al. 2013). This study compared the effects of whole body vibration (40 Hz, 0.83 mm), field-based agility re-warm-up, and seated rest on maximal voluntary torque in semi-professional soccer players (*n* = 10, age 20) (Table 1). The players received these treatments during half time of a simulated soccer match to determine if whole body vibration compared with the control treatments (i.e., field-based agility re-warm-up, and seated rest) could reduce torque loss more effectively upon return to the field for the second half. Over the 90-min simulated game, peak torque declined (*p* < 0.01). However, between the end of the first half and start of the second half, the most critical period, when whole body vibration was applied, maximal quadriceps concentric torque decreased by 6.4 % (whole body vibration: 238.3 vs. 223.1 Nm), remained unchanged after agility re-warm-up (0.4 %, active control), and increased by 4.3 % after seated rest (passive control). These data suggest no sparing effect of whole body vibration on maximal voluntary torque under these conditions. The authors’ broader analyses also showed no statistical differences between whole body vibration and the agility (active) control treatment. The effect size of −0.43 and −0.36 for the comparison between treatment vs. active (agility) and treatment vs. passive control (seated rest) suggests low practical sparing effect on maximal voluntary concentric torque.

#### Chronic effects

**Fig. 2:**
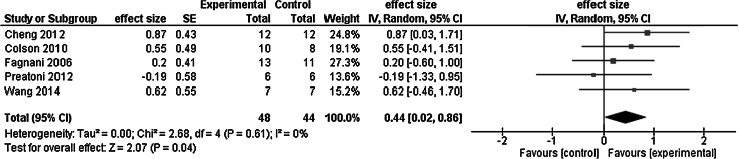
Meta-analysis of the chronic effects of whole body vibration training on maximal voluntary leg force in competitive and/or elite athletes

We have identified five studies that examined the chronic effects of whole body vibration on maximal voluntary force. Whole body vibration was delivered at a frequency of 32.0 Hz (range 25–40), amplitude of 3.9 mm (range 1.5–6.0), and acceleration of 167.2 (range 53–290) and administered over an average of 6.0 weeks (range 4–8) in 58 male and 47 female competitive and/or elite athletes (*N* = 105; basketball, soccer, softball, tennis, and volleyball players, gymnasts, sprinters, taekwondo athletes) (Cheng et al. 2012; Colson et al. 2010; Fagnani et al. 2006; Preatoni et al. 2012; Wang et al. 2014). These studies measured maximal voluntary force, torque, and rate of force development on an isokinetic dynamometer and 3RM leg strength with free weights (Table 2). The average increase in maximal strength was 14.6 % when athletes performed the training exercises in combination with whole body vibration and it was 10.8 % when they received whole body vibration only. Active and passive control interventions, respectively, increased maximal strength by 4.5 and 6.8 %. Thus, whole body vibration combined with exercise compared with control had a net effect of 10.2 % on maximal voluntary force. Figure 2 shows the meta-analysis and suggests that this 10.2 % net effect corresponds to a small weighted mean effect size of 0.44 (0.22 and 0.73, respectively, compared with active and passive control). The net effect of whole body vibration plus exercise vs. whole body vibration alone was 3.8 % (effect size = 0.11) and it was 7.7 % vs. passive control (effect size = 0.73).

Whole body vibration treatment significantly increased maximal voluntary force in three of the four studies (Cheng et al. 2012; Fagnani et al. 2006; Wang et al. 2014). Eight weeks of whole body vibration in squat position improved maximal rate of tension development by 17.0 % compared with the 14.1 % increase produced unloaded squats without whole body vibration (group by time interaction, *p* < 0.05) (Cheng et al. 2012). However, there was over 200 N/ms difference in rate of tension development at baseline between the two groups. Four weeks of static strength training at 75 % of standing 1RM combined with whole body vibration improved national caliber sprinters’ maximal voluntary isometric knee extension torque by 21 % (*p* < 0.05) while the 10.4 and 1.5 % improvements after training with whole body vibration alone or strength training alone at 75 % of standing 1RM were not significant (group by time interaction, *p* < 0.05) (Wang et al. 2014). In this study also there was as much difference in mass-normalized torque at baseline (~0.3 Nm/kg) as there were increases in the whole body vibration-only control group. When competitive volleyball players, basketball players, track and field athletes, and gymnasts performed their event-specific exercises on a whole body vibration platform, 3RM leg press increased by 12.7 % or 48–426 kg (*p* < 0.05) compared with the 10 kg gain (n.s.) in passive control group (Fagnani et al. 2006). However, the control vs. the intervention group’s initial 3RM was 16 kg higher. In contrast to these positive adaptations, strength training both with and without whole body vibration improved national level soccer and softball players’ maximal voluntary isometric knee extension force by 10 % (n.s.) (Preatoni et al. 2012). There was also only 5 and 0 % increase in isometric knee extension maximal voluntary force after 12 sessions of squat exercise with and without whole body vibration in a group of competitive basketball players (Colson et al. 2010). In these five studies, the mean improvements were 0.79 % per session in the intervention groups (range 0.41–1.75) and 0.27 % per session in the control groups (range −0.04 to 0.59). Taken together, there is inconsistent evidence that chronic exposure to whole body vibration in up to 16 sessions delivered over up to 8 weeks would reliably and functionally meaningfully increase maximal voluntary leg muscle force in diverse groups of athletes.

### Effects of whole body vibration on maximal power generation in athletes

#### Acute effects

We have identified six studies that determined the acute effects of whole body vibration at an average frequency of 33.5 Hz (range 26–40), amplitude of 3.1 mm (range 0.8–6.0), and acceleration of 124.1 m/s^2^ (range 52.4–193.2) on a direct or a proxy measure of leg power in competitive and/or elite athletes (Bullock et al. 2008; Cochrane and Stannard 2005; Despina et al. 2014; Lovell et al. 2013; Naclerio et al. 2014; Rhea and Kenn 2009). These studies included 42 male and 35 female (*N* = 77) athletes ranging in experience between Division I US College and Olympic levels, representing sports such as field hockey, Australian football, rhythmic and standard gymnastics, soccer, football, baseball, track and field, and power lifting. Before and after short-term whole body vibration and control interventions, leg power was measured during an unloaded countermovement vertical jump in the form of mechanical power or jump height and also as mechanical power measured during squats at a load of 75 % of 1RM.

**Fig. 3:**
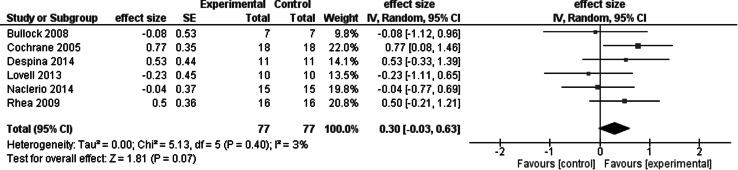
Meta-analysis of the acute effects of whole body vibration on leg power in competitive and/or elite athletes

Figure 3 shows the meta-analysis of the acute effects of whole body vibration on leg power in competitive and/or elite athletes. Compared with control interventions, whole body vibration had minimal effects on leg power signified by the weighted mean effect size of 0.30. Compared with control, when athletes performed various squat maneuvers combined with whole body vibration, leg power increased by 4.7 %. The largest effect corresponded to a medium effect size of 0.77 so that field hockey players’ vertical jump height increased 8.1 % after the whole body vibration intervention compared with the −3.0 and −0.3 % change after cycling active and no intervention passive control (group by time interaction, *p* < 0.05) (Cochrane and Stannard 2005). We must note that the observed small reductions in jump power after the cycling control treatment is not unexpected because low cadence cycling, i.e., 50 RPM, can have unfavorable effects on jumping performance (Marquez et al. 2009). When ten semi-pro soccer players performed whole body vibration and agility re-warm-up during the half time of a simulated soccer match, the effects of the two treatments were negligible and similar (−1.5 and 1.0 %) (Lovell et al. 2013). Whole body vibration had the largest acute effect on leg power in 15 Division I American college football players. Together, the data suggest minimal or no immediate effect of whole body vibration on leg power measured directly or indirectly in competitive and/or elite athletes.

#### Chronic effects

The search has identified nine studies that examined the chronic effects of whole body vibration at an average frequency of 32.8 Hz (range 25–40), amplitude of 4.8 mm (range 4.0–8.0), acceleration of 208.4 m/s^2^ (range 98.6–386.5) over an average of 11.1 weeks (range 4–15) in 33 male and 150 female (*N* = 183) on leg power in basketball, rugby, soccer, softball, and volleyball players, dancers, gymnasts, and sprinters (Annino et al. 2007; Colson et al. 2010; Fagnani et al. 2006; Fernandez-Rio et al. 2010, 2012; Fort et al. 2012; Marshall and Wyon 2012; Preatoni et al. 2012; Suarez-Arrones et al. 2014). Exercise training included, with or without whole body vibration, loaded and unloaded squat exercises, practice of discipline skills, and strength training. Leg power was measured before and after these interventions during a countermovement vertical jump (in milliseconds, cm), countermovement jumps performed for 15 or 30 s, and various forms of squat and leg press exercises (Table 2).

**Fig. 4:**
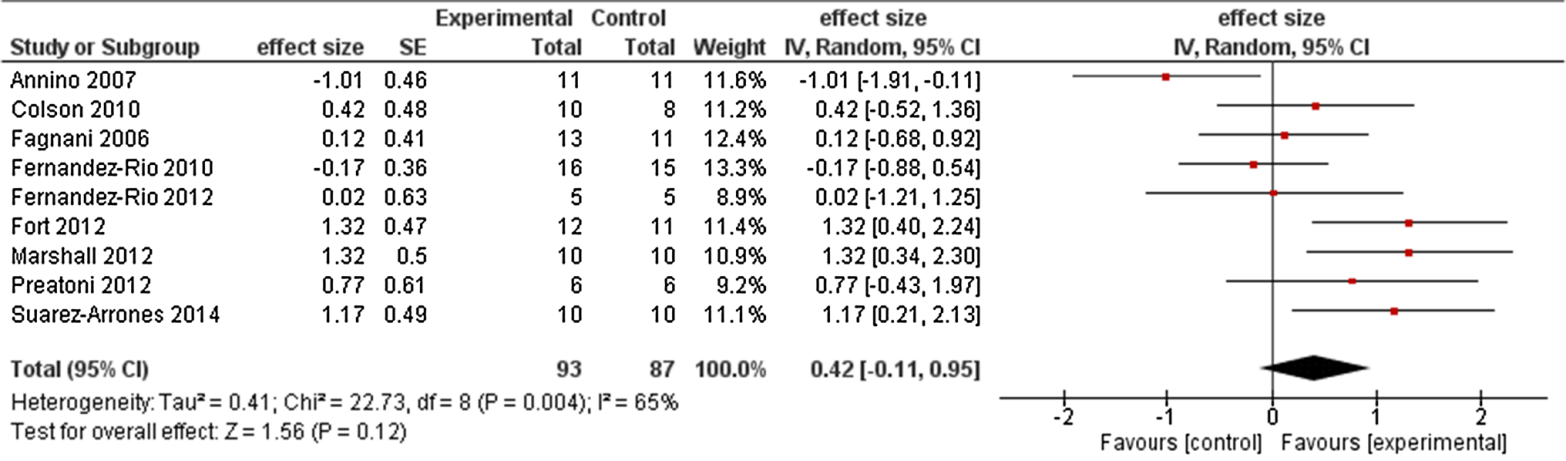
Meta-analysis of the chronic effects of whole body vibration on leg power in competitive and/or elite athletes

Figure 4 shows the meta-analysis of the chronic effects of whole body vibration on leg power in competitive and/or elite athletes. When whole body vibration was used alone, the effect was 1.0 %. Active and passive control produced, respectively, 4.4 and 9.6 % change in leg power. The net effect of exercise plus whole body vibration vs. control was 9.7 % (weighted mean effect size = 0.42).

Three of the nine studies revealed favorable chronic effects of whole body vibration on athletes’ leg power. A 15-week exercise training consisting of squat, step, and jump movements on the whole body vibration platform (30 Hz, 4 mm) improved competitive basketball players countermovement vertical jump height by 2.5 cm or 10 % more than the active control group that performed the same movements without whole body vibration (group by time interaction, *p* < 0.05) (Fort et al. 2012). A 4-week-long program made up of dance movements performed on the whole body vibration platform (35 Hz, 8 mm) improved competitive dancers’ countermovement vertical jump height 3.0 cm or 7 % more than the active control group (group by time interaction, *p* < 0.05) (Marshall and Wyon 2012). An 8-week whole body vibration (25 Hz, 4 mm) program during which various elite athletes performed discipline-specific skills improved countermovement jump height by 1.8 cm or 6.3 % more compared with the changes in the active control group (group by time interaction, *p* < 0.05) (Fagnani et al. 2006). In eight of the nine studies, it was possible to compute the improvements per session. In these eight studies, the mean improvements were 0.96 % per session in the intervention groups (range 0.06–1.75) and 0.61 % per session in the control groups (range −0.39 to 1.75).

However, a careful analysis of the data revealed several inconsistencies that warrant caution in interpreting the weighted mean effect size of 0.42 (Fig. 4, Table 2). Although 24 sessions of squat exercises with whole body vibration compared with the active control group had an 8.6 % net effect on leg power in dancers (*p* < 0.05, *t* test and Bonferroni adjusted) (Annino et al. 2007), the initial difference in leg power measured during a leg press against 100 kg mass was 81 W lower in the whole body vibration group, a baseline difference that is 1.7 times greater than 49 W increase produced by the whole body vibration intervention, resulting in a −1.1 effect size (Annino et al. 2007). There was also a large, 3.8 cm, difference at baseline in the countermovement jump height, which was 1.4 cm greater than the 2.4 cm change caused by whole body vibration in the 8-week whole body vibration program followed by dancers (Marshall and Wyon 2012). Finally, countermovement jump height was actually numerically almost identical in the whole body vibration (31.9 cm) and in the active control group (31.3 cm) in the study that examined the effects of training with discipline-specific skills (Fagnani et al. 2006). In total, the results from these nine studies provide little and inconsistent evidence for whole body vibration alone or in combination with exercise, to increase competitive and/or elite athletes’ leg power.

### Effects of whole body vibration on flexibility in athletes

#### Acute effects

We have identified two studies that examined the acute effects of whole body vibration at a frequency of 28.0 Hz (range 26–30), amplitude of 4.0 mm (range 2.0–6.0), and acceleration of 115.5 m/s^2^ (range 71.0–160.0) on flexibility (Cochrane and Stannard 2005; Despina et al. 2014). These two studies included 29 elite and Olympic female field hockey players and rhythmic gymnasts and measured flexibility using the sit-and-reach test before and after short-term whole body vibration interventions (Table 1).

Standing on the vibration platform in a semi-squat position for a few bouts increased elite field hockey players’ range of motion by 8.3 % while the change was 5.6 and 5.3 % after active (cycling) and passive control (Cochrane and Stannard 2005). Although the group by time interaction was significant, note that the difference between whole body vibration and active control (cycling) in sit-and-reach performance was only 1.3 cm after the two interventions, corresponding to a small effect size of 0.22 for the interaction term. There was virtually no effect (1 %) of whole body vibration performed in the squat positions by Olympic level rhythmic gymnasts’ flexibility (Despina et al. 2014). Additional studies are needed to determine if whole body vibration alone or in combination with exercise can acutely improve athletes’ flexibility.

#### Chronic effects

We have identified two studies that examined the chronic effects of whole body vibration at an average frequency of 30.0 Hz (range 25–35), amplitude of 6.0 mm (range 4.0–8.0), and acceleration of 242.5 m/s^2^ (range 98.6–386.5) over 6 weeks (range 4–8) in 41 female competitive and elite athletes (Fagnani et al. 2006; Marshall and Wyon 2012) (Table 2). Dancers, volleyball and basketball players, track and field athletes, and gymnasts practiced their discipline-specific movements with whole body vibration and in the active control condition without whole body vibration. Exercise training included, with or without whole body vibration, a practice of discipline movements (half squats, loaded and unloaded squat exercises) and strength training. Flexibility was measured using the sit-and-reach test (Fagnani et al. 2006) or by measuring the active hip range of motion in a specific dance position (Marshall and Wyon 2012).

Both studies revealed statistically significant chronic effects of whole body vibration on flexibility. The increase in sit-and-reach was 3.0 cm or 15.3 % compared with the active control (1.2 cm or 6.5 %) so that the post-intervention values also revealed a 3.0 cm between-group difference with a medium effect size of 0.61 (group by time interaction) (Fagnani et al. 2006). However, the authors used *t* tests instead of analysis of variance. Whole body vibration also improved competitive dancers’ hip range of motion to 117.5° from 99.9° (*p* < 0.05) with no change in active controls (1.8° or 1.7 %, n.s.), producing a medium effect size of 0.57. However, these results may be affected by the large, 5.4°, between-group differences in range of motion at baseline (whole body vibration: 99.9°, active control: 105.3°). In these two studies, the mean improvements were 1.42 % per session in the intervention groups (range 0.64–2.20) and 0.24 % per session in the control groups (range 0.21–0.27). Although these data suggest a consistent chronic effect of whole body vibration on joint flexibility, future studies will have to confirm the sporadic preliminary evidence.

### Effects of whole body vibration on athletic performance in athletes

#### Acute effects

We have identified six studies that determined the acute effects of whole body vibration at an average frequency 33.5 Hz (range 26.0–45.0), amplitude of 2.8 mm (range 0.8–6.0), and acceleration of 109.0 m/s^2^ (range 52.4–175.7) on athletic performance in competitive and/or elite athletes (Bullock et al. 2008; Cochrane 2013; Despina et al. 2014; Guggenheimer et al. 2009; Lovell et al. 2013; Padulo et al. 2014). These studies included 51 male and 24 female (*N* = 75) athletes ranging in experience from Division I US College to Olympic levels, representing a variety of disciplines (rhythmic gymnastics, netball, skeleton, soccer, track and field). The acute interventions included the execution of gymnastics movements, high-knee running, and squats on the whole body vibration or shuttle runs were supplemented with whole body vibration (Table 1). Active control conditions included the same (i.e., squats, dry batting, etc.) or other (i.e., agility) exercises and passive control involved rest. We expressed the data so that improvements in performance (i.e., decrease in sprint time, increase in velocity) were set to positive percent changes.

**Fig. 5:**
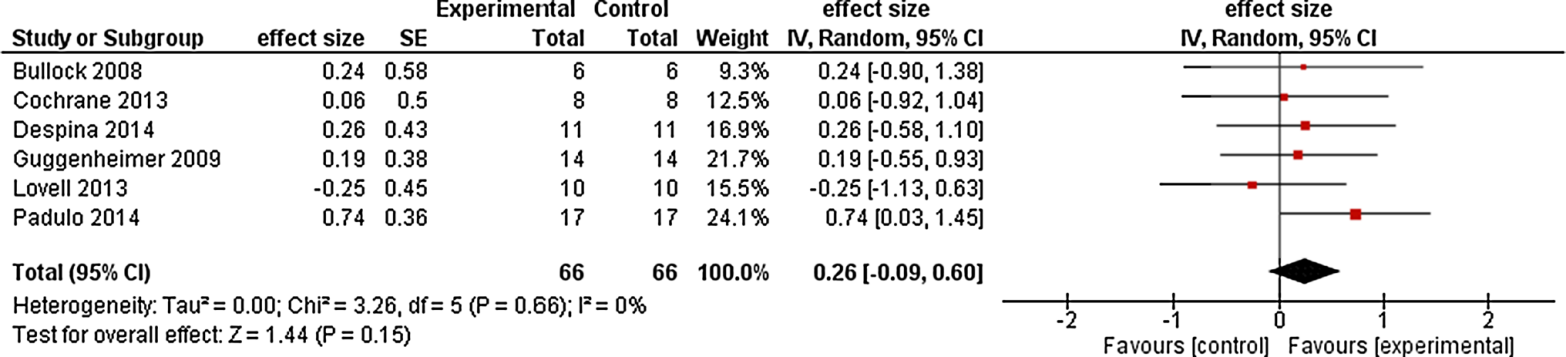
Meta-analysis of the acute effects of whole body vibration on athletic performance in competitive and/or elite athletes

Figure 5 shows the meta-analysis of the effects of whole body vibration on athletic performance, with a weighted mean small effect size of 0.26. Considering the six studies, the acute effects of whole body vibration alone or added to specific activity were minimal. None of the studies reported a significant group by time interaction. Whole body vibration combined with exercise improved athletic performance −1.9 % while whole body vibration alone had a −1.0 % effect. Active and passive control, respectively, had −3.3 and −1.8 % effect. For example, Olympic caliber skeleton athletes’ 30 m sprint time was 4.18 s before and 4.21 s after (n.s.) bouts of unloaded half squats done on the whole body vibration (effect size = 0.24). Premier level netball player’s reactive agility run times were numerically almost identical with and without whole body vibration (1.90 and 1.92 s, n.s.; effect size = 0.06). Whole body vibration also had no effect on Olympic level rhythmic gymnasts’ weight shifting performance, as the post-treatment values were similar: 84.0 % after whole body vibration and 82.5 % after gymnastics movements performed without whole body vibration (n.s., effect size = 0.26). Taken together, there is little and inconsistent evidence that whole body vibration would acutely increase athletic performance or its proxies in athletes.

#### Chronic effects

The search has identified five studies that examined the chronic effects of whole body vibration at a frequency of 31.0 Hz (range 30.0–35.0), amplitude of 3.1 mm (range 1.5–4.0), and acceleration of 115.2 m/s^2^ (range 53.2–142.0) on athletic performance in 78 male and 30 female (*N* = 108) competitive and/or elite athletes (Cheng et al. 2012; Delecluse et al. 2005; Fort et al. 2012; Suarez-Arrones et al. 2014; Wang et al. 2014). Over an average of 7.6 weeks, competitive and/or elite basketball, rugby, tennis, and volleyball players, and sprint, and taekwondo athletes performed interventions consisting of various loaded and unloaded squat movements on or off the whole body vibration platform or whole body vibration was added to sprint and shuttle run training. Measurements included running economy, 20–30 m sprints, and balancing on one leg (Table 2).

**Fig. 6:**
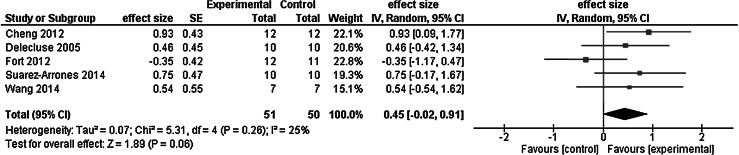
Meta-analysis of the chronic effects of whole body vibration on athletic performance in competitive and/or elite athletes

Figure 6 sows the meta-analysis of the chronic effects of whole body vibration on athletic performance in competitive and/or elite athletes. The weighted mean effect size was small, 0.45. Whole body vibration added to exercise training and whole body vibration alone improved athletic performance, respectively, by −1.2 and 2 %. Active and passive control interventions improved performance, respectively, 3.5 and 1.9 %. The net effect of whole body vibration combined with exercise training was thus 3.2 % and the net effect of whole body vibration when used alone was −4.7 % relative to active control.

Two of the five studies reported a significant group by time interaction. Eight weeks of whole body vibration (30 Hz, 1.5 mm) in the unloaded half-squat position improved running economy by 7.7 % compared with the 1.9 % gain after the passive control intervention (group by time interaction, *p* < 0.05; effect size = 0.93). Note, however, that the absolute gain was only 0.3 m/ml/kg (see Discussion for further analysis of this finding) (Cheng et al. 2012). When nationally top ranked sprinters (*n* = 7) received whole body vibration (30 Hz, 4 mm) while they pressed up against an unmovable bar at 75 % of the maximal force in a semi-squat position during a 4-week-long training program, the 30 m sprint velocity increased by 0.16 m/s or 2.3 % to 7.01 from 6.85 m/s (*p* < 0.05) (Wang et al. 2014). However, when a second small group of athletes (*n* = 7) received whole body vibration only without muscle contraction, 30-m sprint velocity actually *decreased* by −0.25 m/s or 3.8 % to 6.37 from 6.62 m/s (*p* < 0.05, effect size = 0.89), causing the group by time interaction. Exercise training with 75 % MVC only (no whole body vibration) did not affect running velocity (pre: 6.72 vs. post: 6.75 m/s, n.s.). The remaining three studies showed no significant effects, with 15 weeks of squat, step, and jump program actually worsening elite basketball players’ one-legged balance performance by 10.9 % (n.s.; effect size = -0.35). When elite sprinters trained for 5 weeks with and without whole body vibration (35 Hz, 2 mm), the start time for 30 m sprint did not change (364.3 ms both pre and post with whole body vibration; pre: 374.2 ms, post: 375.2 ms without whole body vibration) (Delecluse et al. 2005). In four of the five studies, it was possible to compute the gains per session. In these four studies, the mean improvements were 0.17 % per session in the intervention groups (range −0.31 to 0.63) and again 0.17 % per session in the control groups (range 0.02 to 0.42). Taken together, there is limited and inconsistent evidence for whole body vibration to improve athletic performance in athletes.

## Discussion

Results of the present review support the prediction that whole body vibration has little or no acute and chronic effects on competitive and/or elite athletes’ athletic performance. Whole body vibration combined with exercise had an overall 0.3 % *acute* effect on maximal voluntary force (−6.4 %, effect size = −0.43, 1 study), power (4.7 %, weighted mean effect size = 0.30, 6 studies), flexibility (4.6 %, effect size = −0.12 to 0.22, 2 studies), and athletic performance (−1.9 %, weighted mean effect size = 0.26, 6 studies) in 191 (103 male, 88 female) athletes representing eight sports. Exercise without whole body vibration produced a −1.0 % acute change in the four categories of outcome measures. Compared with active controls, whole body vibration combined with exercise and whole body vibration alone had a *net acute* effect of −0.9 and 0.6 %. By combining the data from the meta-analyses in Figs. 3 and 5, whole body vibration had an overall small acute effect on athletic performance as quantified by the effect size of 0.28.

Whole body vibration combined with exercise had an overall 10.2 % *chronic* effect on maximal voluntary force (14.6 %, weighted mean effect size = 0.44, 5 studies), leg power (10.7 %, weighted mean effect size = 0.42, 9 studies), flexibility (16.5 %, effect size = 0.57 to 0.61, 2 studies), and athletic performance (−1.2 %, weighted mean effect size = 0.45, 5 studies). Whole body vibration administered without being added to exercise produced 4.6 % chronic change in the four categories of outcome measures. Compared with active control, whole body vibration combined with exercise and whole body vibration alone had a *net chronic* effect of 4.2 and 6.7 %. By combining the data from the meta-analyses in Figs. 2, 4, and 6, whole body vibration had an overall small chronic effect on athletic performance, as quantified by the effect size of 0.44. By combining acute and chronic studies, the overall effect size was 0.36 (Figs. 2, 3, 4, 5, 6). On the whole, the quality of studies was low as the average PEDro scores for the acute and chronic studies were below the threshold of 6.0 (Table 3). We interpret these data to mean that whole body vibration has small and inconsistent acute and chronic effects on athletic performance or its proxy measures in competitive and/or elite athletes.

### Acute effects

Previous reviews used qualitative analyses instead of a meta-analysis to quantify the acute and chronic effects of whole body vibration on athletic performance in competitive and/or elite athletes. In addition, a previous review addressed the topic as ‘whole body vibration in sport’ but its conclusions are probably not relevant to competitive and/or elite athletes due to the inclusion of non-elite athletic groups as subjects (‘physical education students’, ‘recreational athletes’, ‘former high class kayakers’, ‘healthy men’, and ‘untrained subjects’) who received the vibration stimulus through non-standardized formats such as whole body vibration platforms, cables, and other vibrating devices. A sub-analysis in a recent review compared the effects of whole body vibration on performance in athletes vs. sedentary subjects but used vertical jump height as the main outcome, which may have low ecological validity for ‘athletic performance’ (Manimmanakorn et al. 2014).

We expected that whole body vibration alone or added to exercises athletes use in their preparation for competition would increase athletic performance or its proxy measures to a negligible extent because the whole body vibration stimulus represents too small a portion of the total training stimulus in terms of training intensity, duration, and frequency. A previous review also showed that the whole body vibration effect on jump performance was only about one half of the effect size (0.59) observed in untrained healthy adults (0.96). Thus, the whole body vibration effect seems to decrease with increasing training status (Manimmanakorn et al. 2014). That review reported a dose–response effect based on only five studies with an effect size of 0.68 and 0.92 for exposure to whole body vibration shorter or longer than 10 min, respectively (Manimmanakorn et al. 2014). Whole body vibration exposure to 10–20 min per session in relation to practice of specific skills for 1–2 h or 10 % of total duration (Colson et al. 2010) trivializes the whole body vibration effect. We also note that none of the studies addressed the issue whether or not the whole body vibration effect can actually outlast the duration of the session and increase athletic performance or its proxy measures minutes, hours, or days after the original exposure. In the setting of track and field, gymnastics, rhythmic gymnastics, and possibly other sports, it is impossible to receive whole body vibration up to perhaps 30 min before the actual start of the competition and in some sports as in soccer, regulations limit half-time warm-up (Lovell et al. 2013). Therefore, a combination of logistical and physiological factors makes it unlikely that whole body vibration could acutely increase highly trained athletes’ competition performance, a conclusion supported by the net effect of −0.9 and 0.6 % produced by whole body vibration added to exercise and whole body vibration given by itself.

Our analysis, albeit severely limited by relying on only one study (Lovell et al. 2013), revealed actually an unfavorable −6.0 % net effect whole body vibration on maximal force in soccer players (effect size = −0.40). The negative effect of whole body vibration on maximal force was also noted in a previous analytical review but observed mostly in non-elite athletes (Rittweger 2010). Because of the severely limited sample size, the tentative conclusion is that it is probably unlikely that athletes would benefit physiologically or through a motor control mechanism (e.g., warm-up effect, novelty effect, post-activation potentiation) from whole body vibration performed immediately before competition.

### Chronic effects

Compared with control, whole body vibration plus exercise and whole body vibration alone had a net effect on the four outcome measures of 4.2 and 6.7 %, respectively. While these effects seem functionally meaningful, meta-analyses revealed only small practical weighted mean effects: 0.44 (maximal force), 0.42 (power), and 0.45 (athletic performance). A weakness of the analysis is that there were only five chronic intervention studies that examined the effects of whole body vibration on ‘athletic performance’. In this perhaps most relevant category, compared with active control, the net effect of whole body vibration plus exercise and whole body vibration alone was −3.2 and −4.7 %. The outcomes in favor of whole body vibration were small or contradictory. For example, although whole body vibration improved elite runners’ running economy significantly (7.7 %), this improvement in absolute terms was only 0.3 m/ml/kg (Cheng et al. 2012). In this study, running economy was expressed in gross distance and also in gross caloric unit costs. Computed from the authors’ data, running a Marathon in 224.68 min at 3.13 m/s, the average running speed on the treadmill in the study, the improved running economy would have permitted runners to cover 12.73 m longer distance as a result of the vibration treatment. Whole body vibration combined with exercise during which national caliber sprinters pressed against a fixed bar in the squat position at 75 % of maximal force improved 30 m sprint velocity by 0.16 m/s or 2.3 % (*p* < 0.05), the same whole body vibration without the squat exercise actually decreased running speed by 0.25 m/s or 3.8 % (*p* < 0.05). While the second control group, which performed strength training only, did not have an effect on sprint velocity, considering many other studies that reported favorable effects of whole body vibration alone on vertical jump, power, and running performance in non-elite athletes (Issurin 2005; Manimmanakorn et al. 2014; Wilcock et al. 2009) complicates the interpretation of these data (Wang et al. 2014).

Whole body vibration produced the most consistent and statistically significant and functionally meaningful chronic (but not acute) effects on flexibility (12.4 % net effect vs. active control) (Fagnani et al. 2006; Marshall and Wyon 2012). How such an increase in flexibility might occur is unknown. The authors of these studies suggest that vibration acts through an analgesic effect: athletes overcome pain and achieve a greater range of motion. The authors cited studies in which vibration was used to determine the effect of conditioning vibratory stimulation on pain threshold of the human tooth (Ekblom and Hansson 1982) or examined the effects of vibratory stimulation as a pain-relieving agent in patients who suffered from chronic musculoskeletal pain (Lundeberg 1984). These, and many other clinical studies cited in reviews, used 10 or 100 Hz focal vibration to relieve pain, rates whole body vibration studies never use because, for example, 10 Hz can potentially be harmful in the context of whole body vibration (Rittweger 2010). The possibility of a thermic mechanism was also raised, increasing flexibility through increased blood flow (Fagnani et al. 2006; Marshall and Wyon 2012). However, there was actually a greater *decrease* in muscle temperature after whole body vibration compared with agility control during the half time of a simulated soccer match (Lovell et al. 2013). It is also perplexing that the whole body vibration-generated energy associated with the acceleration rapidly dissipates as it progresses from the ankle to hip soft tissue structures (Friesenbichler et al. 2014), yet the significant chronic whole body vibration effect occurred in hip joint flexibility where the whole body vibration effect is the weakest. Thus, how the ~12 % net chronic (but not acute) effects of whole body vibration on range of motion come about remains unknown. More research is needed to elucidate the underlying neuromuscular mechanisms.

### Concluding comments, recommendations, limitations

A large number of whole body vibration studies used ‘passive control’, a comparison the present review tends to de-emphasize. We suggest that the passive control comparisons in research studies are not valid because whole body vibration groups experience extra muscle activation or even perform extra work (as suggested by oxygen uptake measurements), factors that are absent in passive control groups.

Second, most whole body vibration studies in competitive and/or elite athletes were performed off-season. The composition of the off- and in-season training programs is different and whole body vibration may interact differently with the main training stimulus in the two phases. It is also unclear if any positive acute and chronic effects of whole body vibration accrued off-season would actually be maintained and transferred into the in-season phase if the application of whole body vibration at the end of the off-season is withdrawn as athletes move into the in-season and competition phase. Finally, the low efficacy of whole body vibration observed off-season might become even lower when coaches increase in-season training intensity and shift attention to event-specific conditioning.

Third, there is a strong bias in the literature by focusing the use of whole body vibration on physical conditioning without little or no attention to the effects on motor control in competitive/elite athletes. Only one of the 21 studies reviewed here did examine postural adaptations (i.e., weight shift) and balance in professional dancers, quality instead of quantity of performance (Despina et al. 2014). Would whole body vibration compromise or enhance basketball players’ ability to execute jump shots and free throws accurately? How would acute whole body vibration affect a baseball pitcher’s ability to throw the ball accurately and fast? In general, what is the effect of whole body vibration on force control, steadiness, and accuracy? In this context, it has to be clarified whether whole body vibration also produces kinesthetic illusions as reported for single muscle vibrations at high frequencies (Goodwin et al. 1972a, b, c; Taylor and McCloskey 1991).

Fourth, there is a strong need to increase experimental control by including true sham whole body vibration. None of the 21 whole body vibration studies reviewed here used a truly sham control group, resulting in an overestimation of the true whole body vibration effects on athletic performance. Performing whole body vibration with the vibration turned off, as was done in all studies included in the present review, is not a true sham. Sham whole body vibration conceals the mechanical component of vibration. There is documented evidence for truly sham whole body vibration to have a physiological effect, as real and sham whole body vibration resulted in similar changes in bone metabolism with two middle-aged post-menopausal volunteers in the sham group reporting adverse effects (Turner et al. 2011). There was also a significant effect in favor of real vs. sham whole body vibration in only one of ten comparisons (Rogan et al. 2012). Truly sham whole body vibration also produced about one half of the changes in reflex excitability (*p* < 0.05) (Hortobagyi et al. 2014).

Sham or placebo can act through physical, biological, and behavioral mechanisms. Athletes naïve to whole body vibration who, when tested or trained, hear the humming of the motor, see the number on the frequency display, and thus believe that they receive true treatment, while in fact they stand on a platform in which the motors have been detached from the undersurface of the platform, would very likely exhibit strong placebo effects, similar to those reported previously in untrained college students (Hortobagyi et al. 2014). Such an effect due to true sham whole body vibration would thus further minimize the effects of real whole body vibration on motor performance: whole body vibration alone and strength training alone did not improve performance when used as ‘active control’ in dozens of whole body vibration studies, but when whole body vibration was added to strength exercises the performance improved.

We also noticed that several comparisons between whole body vibration and control groups were fraught with methodological problems because: (1) the difference between the initial values in the two groups or conditions was greater than the effects produced by the whole body vibration treatment, favoring the changes in the whole body vibration group; (2) in several studies post-values were actually higher in the control group or control conditions compared with the post-treatment values in the whole body vibration experimental groups; (3) several studies measured multiple outcomes and based the conclusions on the significant effects, omitting the non-significant comparisons; (4) while the changes were statistically significant, functionally they were probably not relevant (i.e., 0.03 s improvement in ice hockey sprint time); (5) several studies used incorrect statistical analyses (*t* tests vs., ANOVAs), and (6) some of these inconsistencies and biases could be the result of having unequal number of males and females, respectively, in the experimental and control groups at baseline. Such issues question the reliability of the findings and the interpretation of the data.

**Fig. 7:**
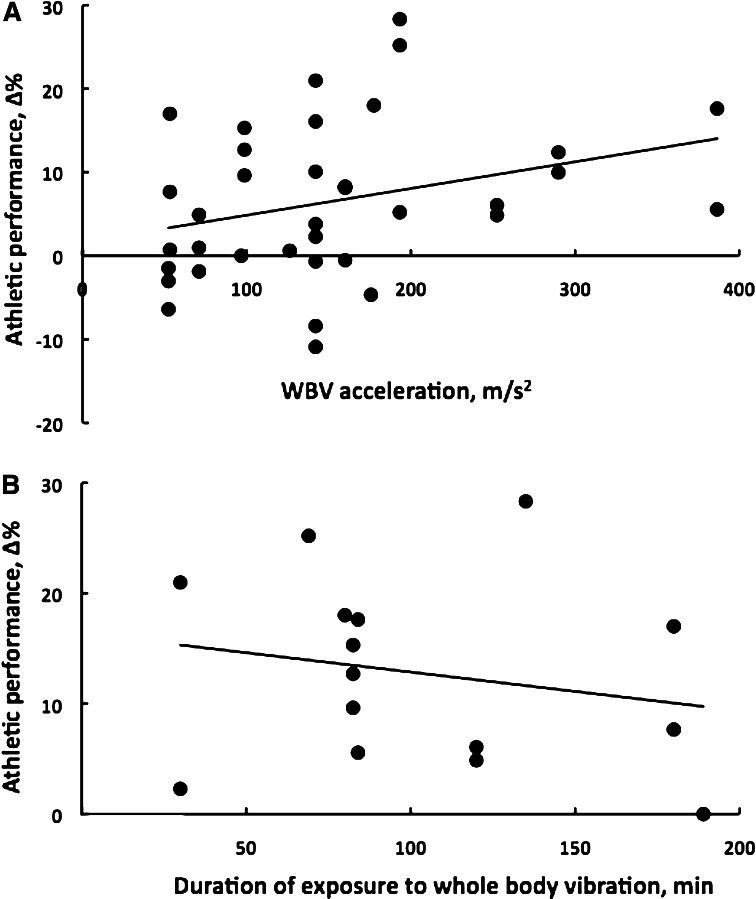
**a** Linear regression of the whole body vibration platform’s acceleration, a summary measure of the whole body vibration training stimulus, on the percent changes this training stimulus induced in athletic performance measured in 36 performance tests in 21 studies that examined the acute and chronic effects of whole body vibration on athletic performance in 272 male and 356 female (*N* = 628) competitive and/or elite athletes. Each symbol corresponds to an acute or a chronic study. There are more than 21 symbols because a given study can have an outcome in as many as three of the four performance domains (i.e., maximal leg force, leg power, flexibility, athletic performance). Because the range of vibration frequency was only 14 Hz (i.e., 26–40), vibration amplitude is the factor that mostly determines the acceleration values. The equations *y* = 0.03*x* + 1.7 and *R* ^2^ = 0.091 (*p* = 0.074) describe the relationship. **b** Linear regression of the total duration of exposure to whole body vibration on the percent changes in athletic performance measured in 15 performance tests in 11 studies that examined the chronic effects of whole body vibration on athletic performance in competitive and/or elite athletes. Each symbol corresponds to a chronic study. There are more than 11 symbols because a given study can have an outcome in as many as three of the four performance domains (i.e., maximal leg force, leg power, flexibility, athletic performance). Studies that did not report the duration of whole body vibration exposure were excluded. The equations *y* = −0.04*x* + 16.4 and *R* ^2^ = 0.043 (*p* = 0.458) describe the relationship

Finally, many studies invoke, recently mostly debunked, ‘neural mechanisms’ to explain the whole body vibration effect on motor performance (Cochrane 2011; Hortobagyi et al. 2014). But what is actually the mechanism that enables conversion of the increases in motor performance measured in a laboratory test task (i.e., vertical jump power) into ‘athletic performance’ (sprinting speed, balance, complex skills)? Under most circumstances, there is a large discrepancy between the nature of the task in which the whole body vibration stimulus is given and the structure of motor skills whole body vibration training intends to improve. There is little or no discussion in these papers how, for example, performing static squats at 75 % of maximal force load would improve running velocity (Wang et al. 2014). Figure 7a illustrates this point by plotting a summary measure of the whole body vibration stimulus, i.e., acceleration of the whole body vibration platform, versus the percent changes induced by the acute and chronic training stimulus in athletes’ performance, revealing a lack of association between the two variables with (*R*^2^ = 0.10, *p* = 0.056) or without (*R*^2^ = 0.091, *p* = 0.074) a log transformation of the acceleration data. There is also no discussion in the experimental studies how the duration of the exposure to vibration being such a trivial fragment of the total training stimulus could meaningfully increase athletic performance. Figure 7b illustrates this point in a crude form by plotting vibration duration against changes in athletic performance in 15 performance measures in 11 chronic vibration studies (*R*^2^ = 0.043, *p* = 0.458; for log transformed duration analysis, *R*^2^ = 0.019, *p* = 0.617). In eight acute studies, the association between percent changes in athletic performance and duration of exposure to vibration based on 14 performance measures was *R*^2^ = 0.029 (*p* = 0.555) and *R*^2^ = 0.00 using the log transformed duration data.

Future studies are encouraged to use only active instead of passive control groups. Future whole body vibration studies should specify the net time and the percent of total time of the whole body vibration stimulus in relation to the total time athletes train. There is a strong need to compare the effects produced by whole body vibration with truly sham whole body vibration. Whole body vibration studies planned for the future should strengthen the randomization process and the inclusion criteria because: (1) the baseline differences between the whole body vibration vs. the control groups are greater than the whole body vibration effects; (2) the post-treatment values are often actually higher in the control than in the whole body vibration group, and (3) effect size computations should consider not only within group but also between group effects at post-intervention, a comparison virtually never reported. An extrapolation of the whole body vibration effects measured off-season to in-season can potentially lead to misleading conclusions because the composition of training and athletes’ states differ vastly at these two time points. Future studies should replicate experiments that did find favorable effects by whole body vibration on performance (for example, Wang et al. 2014). These potential studies should employ systematic protocol modifications with the aim to extract the element of the training stimulus that is responsible for the performance increases. Finally, there is a strong need that future studies consult recommendations published previously concerning the standardization of (1) how the whole body vibration stimulus is delivered (study design, study protocol, parameters) and (2) how the results are reported (Lorenzen et al. 2009; Rauch et al. 2010).

A major limitation of the present review is the low number and quality of studies (Table 3). Specifically, a substantial limitation is that there were only a handful of studies that examined the acute (6) and chronic (5) effects of whole body vibration on ‘athletic performance’ and we were forced to use maximal force and leg power as proxies for ‘athletic performance’, measures that may have low ecological validity for actual performance in the field or on the court. Meta-analysis was originally designed with the intention that results would be pooled from trials using similar methods and dependent variables. Pooling data form studies that used a wide range of vibration platforms, settings, and protocols limits the reliability of the findings, especially from chronic interventions. As in any review, it is possible that certain studies were missed or appeared after the manuscript was completed.

In conclusion, the present review found little and inconsistent evidence that acute and chronic whole body vibration would improve athletic performance in competitive and/or elite athletes. Thus, other types of exercise programs (e.g., resistance training) are recommended if the goal is to improve athletic performance.

## Abbreviations

*A*: Amplitude
*a*: Acceleration
CG: Control group
CMJ: Counter-movement jump
EG: Experimental group
F: Female
*f*: Frequency
Hz: Hertz
M: Male
RM: Repetition maximum
MVC: Maximal voluntary contraction
n.r.: Not reported
n.s.: Not significant
PEDro: Physiotherapy Evidence Database
ROM: Range of motion
SD: Standard deviation
*t*: Duration of exposure to vibration
WK: Week
